# Stent or stoma as a bridge to surgery in patients with acute malignant colorectal obstruction: a comparison of oncologic long-term results

**DOI:** 10.3389/fonc.2026.1777147

**Published:** 2026-04-14

**Authors:** Johannes Asplund, Gabriel Sandblom, Isalee Jallow Göransson, Martin Dahlberg, Åsa Hallqvist Everhov, Göran Heinius

**Affiliations:** 1 Department of Colorectal surgery, Södersjukhuset, Stockholm, Sweden; 2 Department of Clinical Science and Education, Södersjukhuset, Karolinska Institutet SÖS, Stockholm, Sweden

**Keywords:** colorectal obstruction, oncologic outcome, self-expanding metallic stents, SEMS, stent, stoma

## Abstract

**Purpose:**

To evaluate the long-term oncologic results in patients treated for malignant left-sided colorectal obstruction with colonic stent or a deviating stoma as bridge to surgery.

**Methods:**

This was a single-center, retrospective cohort study comparing survival between 24 patients treated with stents for acute left-sided malignant obstruction and 32 patients who underwent a diverting stoma from 2003 to 2014. All patients subsequently underwent resection surgery with curative intent.

**Results:**

There were no significant differences between the two groups regarding age, sex, ASA classification, adjuvant chemotherapy, or tumor stage. Median time from acute treatment of obstruction to resection surgery was 30 days in the stent group and 55 days in the stoma group. There were no differences in 30-day complications between the two groups, neither for complications related to the stent or stoma intervention nor for overall complications. Stent-related perforation rate was 1/24 (4.2%). The cumulative 5-year overall survival was 63% in patients treated with a stent and 77% in patients treated with a stoma (p = 0.319). There were two cases of local recurrence in each group (p = 1.0).

**Conclusions:**

We found no differences in short-term complications or long-term oncologic outcomes between the two treatment groups. However, this may be due to a type II error, and larger studies are needed to confirm these results.

## Introduction

Colorectal cancer is the third most common cancer worldwide, with high incidence as well as mortality (1). Acute obstruction is the first symptom in up to 20% of patients with newly diagnosed colorectal cancer, most commonly due to left-sided tumors (2). Surgery is the standard treatment and may be performed with or without resection of the tumor, primary anastomosis, or a diverting stoma. Emergency resection is, however, associated with higher mortality (3, 4), more complications (4), and lower cancer-specific survival compared to those who undergo planned surgery (5). Placement of colorectal self-expanding metallic stents rapidly alleviates obstruction in >90% of cases and has become an attractive treatment option for high-risk patients and those with locally advanced tumors (6). Studies comparing stent insertion with emergency resection suggest shorter hospital stays (7–9), a reduced need for a stoma, and lower morbidity and mortality after stent insertion (10–14). In particular, stents are preferred for palliative treatment of patients with malignant colorectal obstruction (15).

Stenting may also be helpful as a bridge to surgery by alleviating the acute symptoms, allowing time for recovery and optimization before definitive surgery (8, 9). Stent as bridge to surgery has been controversial in patients with potentially curable disease (16, 17) and was not recommended in the European Society of Gastrointestinal Endoscopy (ESGE) guidelines in 2014. However, in the updated ESGE guidelines in 2020, use of stent as a bridge to surgery is considered a valid alternative for left-sided malignant colonic obstruction (18). The long-term oncological outcome from stenting has not been extensively studied, and there have been indications of hematolymphogenic spread of cancer cells after colonic stenting (19) and perforations that could cause intraperitoneal dissemination of the tumor (20). Perforations may be evident in some cases, but there could also be occult perforations that remain unnoticed but lead to upstaging of the disease.

Stents were used in Sweden as a bridge to surgery mainly until 2011 when two ongoing randomized studies comparing emergency stenting to surgery were suspended prematurely due to colon perforations in the stent group (21, 22). Since then, patients with acute left-sided obstruction have usually been treated with a temporary deviating stoma before resection surgery, and the use of SEMS in Sweden for patients without metastasis has now declined to only 2–5 patients per year, compared to 18–29 per year during the period 2007-2009 (23). The aim of this study was to evaluate the short-term complication rate and long-term oncologic outcomes in patients with acute malignant colonic obstruction who were treated with stent insertion (primarily before 2011) as a bridge to surgery, compared with those treated with a diverting stoma (primarily after 2011).

## Methods

### Setting

This study was conducted at the Stockholm South General Hospital, a public general emergency hospital with Sweden’s largest emergency ward, serving a population of about 700,000 individuals. Colonic stents were inserted by experienced endoscopists using image guidance. The choice to use a stent to treat the obstruction was, until 2011, largely determined by the availability of an endoscopist (both on call and during daytime hours).

### Data sources

Patients treated with stent insertion were identified in a local database, including all patients treated from 2003 to 2005, and in KVALOG, a local quality register for colorectal cancer established in 2006. KVALOG holds individual patient data, including dates of diagnosis and treatment. All patients treated with stoma were identified in KVALOG. Patient data for all participants were validated by reviewing the patient charts.

### Study design

This was a retrospective cohort study comparing outcomes for patients treated with stent insertion (exposed) or deviating stoma (comparison group).

### Selection of patients

**Figure 1:**
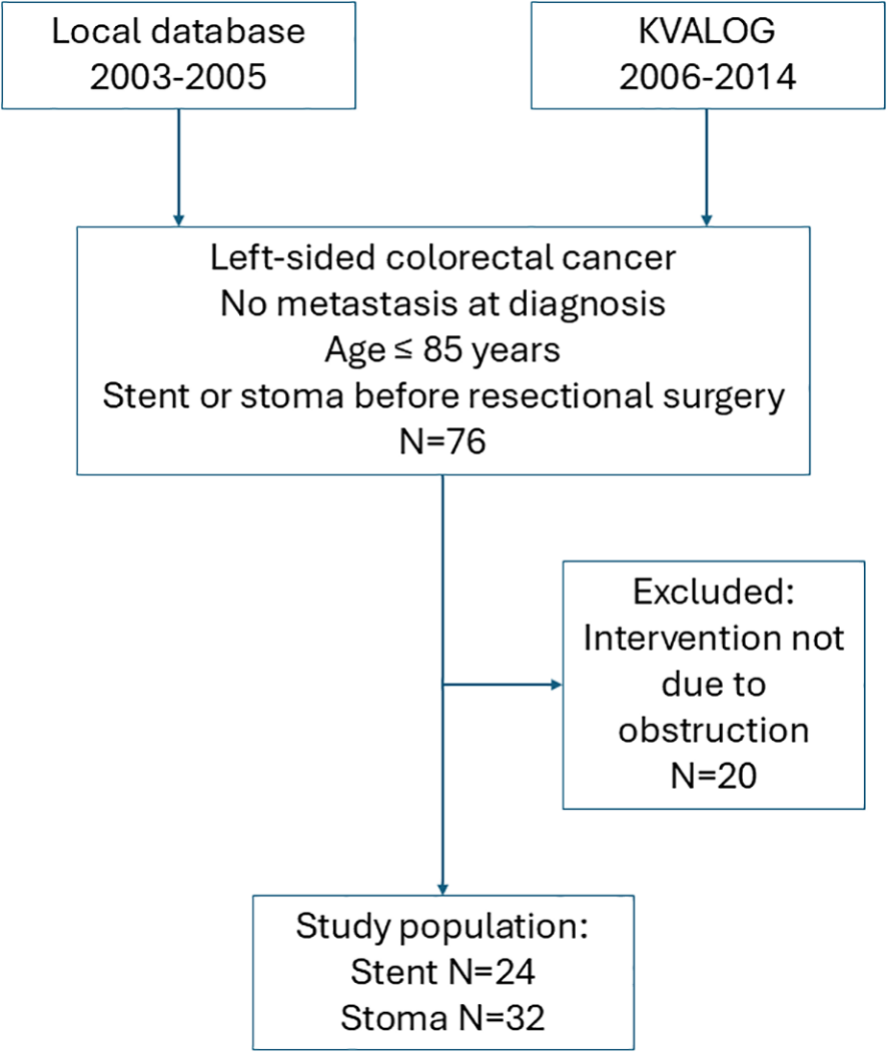
Flowchart of patient inclusion, exclusions, and final groups in the analysis.

**Figure 2:**
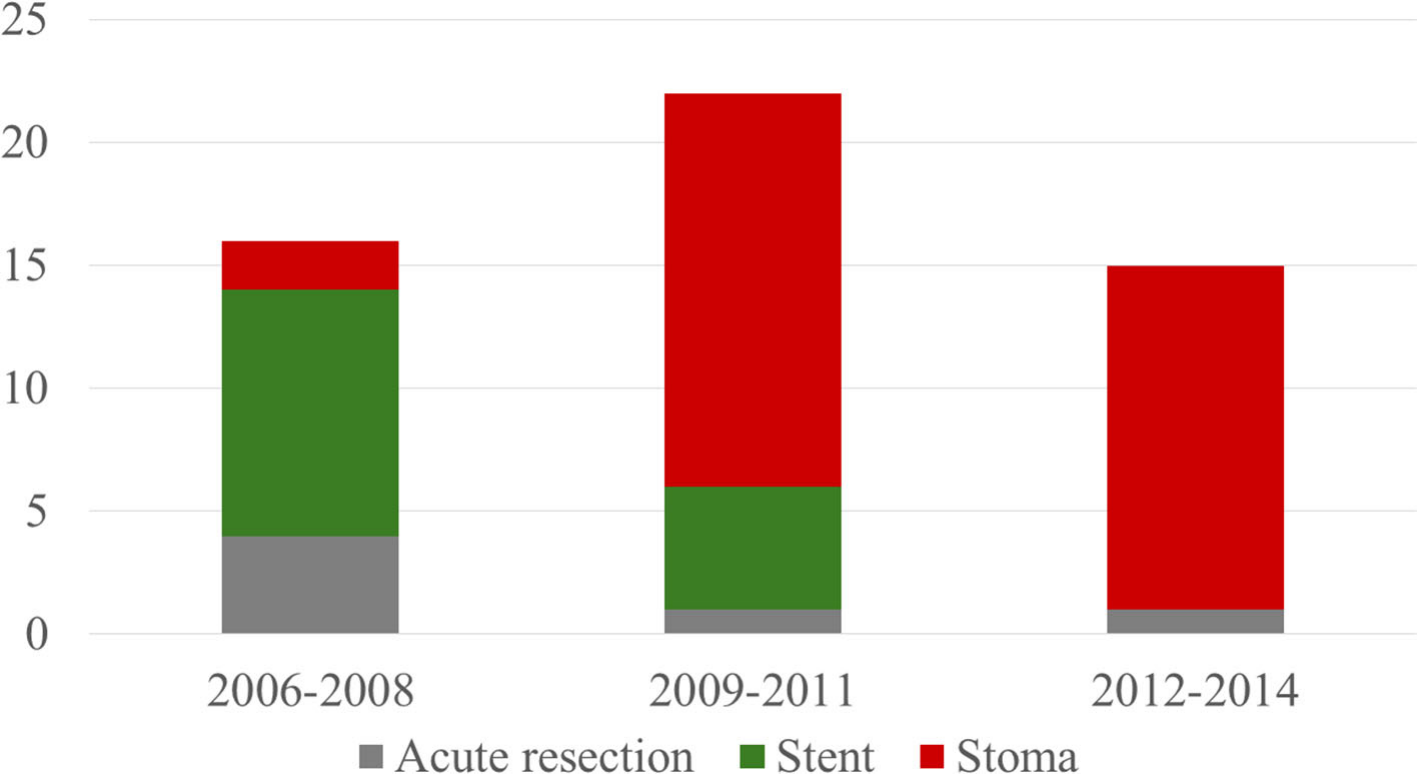
Distribution of treatment during the years of study. All patients admitted aged ≤85 years with acute malignant obstruction of the left part of colon and rectum without confirmed metastases (M0).

We identified all patients aged ≤ 85 years with acute colorectal obstruction due to left-sided (in or distal to the splenic flexure) colorectal cancer but no metastases at diagnosis, treated with stent insertion as a bridge to resectional surgery, 2003-2012 (n = 24, **Figures 1**, **2**).

### Comparison group

As a comparison group, we selected patients aged ≤ 85 years with acute colorectal obstruction due to left-sided colorectal cancer but no metastases at diagnosis treated with a diverting stoma as a bridge to resectional surgery, 2006-2014 (n = 32, **Figure 2**).

### Clinical outcomes

Date and type of cancer recurrence, last follow-up, and date of death were retrieved from the electronic patient chart that is synchronized with the Swedish population register.

In the estimation of overall survival, patients were followed from the date of resection to the date of death or the last follow-up (September 2025).

In the estimation of recurrence-free survival, the patients were followed from resection surgery to the date of cancer recurrence, death, or last follow-up. Technical success was defined as stent placed correctly across the stricture and clinical success defined as decompression of the colon and relief of obstructing symptoms.

### Covariates

Covariates were age, sex, year, ASA (American Society of Anesthesiologists) classification, tumor location, use of adjuvant chemotherapy, and tumor stage.

### Statistical analysis

Continuous variables were compared with the Mann-Whitney U-test and categorical variables with the Chi-square or Fisher´s exact test. Survival analysis was performed using the Kaplan-Meier method, and overall and recurrence-free survival in patients treated with stent or stoma were compared by the log-rank test. We used Cox regression to estimate crude and adjusted hazard ratios (HR) with 95% confidence interval (CI) for overall and recurrence-free survival. The data was analyzed using IBM SPSS 30.0, with p < 0.05 considered statistically significant.

### Ethical statement

The study was approved by the regional Ethical Review Board in Stockholm, Sweden (diary number 2017/1723-31/1 and 2025-05117-01).

## Results

### Population characteristics

The median age was 72 years in the stent group as well as the stoma group. There were no significant differences between the two groups regarding gender, ASA-classification, adjuvant chemotherapy or tumor stage (**Table 1**). The majority of patients in the stent group were treated before 2011, and the majority of patients in the stoma group treated after 2010 (**Figure 2**). The most common tumor location was in the sigmoid colon. The median follow-up time was 18.5 years in the stent group and 14 years in the stoma group. Median time from initial treatment to resection surgery was 30 days in the stent group and 55 days in the stoma group (p = 0.001). The diagnosis of colorectal obstruction was based on abdominal CT-scan in all cases, and all patients experienced symptoms typical of intestinal obstruction. Hanaro stent was used in 23 patients and WallFlex in one. In the stoma group, all patients were treated with a diverting loop colostomy, either sigmoid or transverse. The stoma surgery was performed by a colorectal specialist when available; otherwise, by a general surgeon.

**Table 1:** Characteristics of patients treated with stent or diverting stoma.

	Stent	Stoma	p-value
	N=24	N=32	
**Age (years)**			0.72
median(min-max)	72 (47-83)	72 (38-83)	
**Sex n (%)**			0.42
Male	15 (62)	18 (56)	
**Year n (%)**			**<0.001**
2003-2006	13 (54)	1 (3)	
2007-2010	9 (38)	12 (38)	
2011-2014	2 (8)	19 (59)	
**ASA-classification n (%)**			0.19
I-II	15 (62)	15 (47)	
III-IV	9 (38)	17 (53)	
**Tumor location n (%)**			0.59
Splenic flexure	4 (17)	4 (12)	
Descending colon	8 (33)	10 (31)	
Sigmoid colon	10 (42)	12 (38)	
Rectum	2 (8)	6 (19)	
**Complication related to stent or stoma intervention (30-day Clavien-Dindo) n (%)**			0.85
1	0 (0)	1 (3)	
2	3 (8)	3 (9)	
3a	1 (4)	2 (6)	
3b	3 (12)	0 (0)	
4	0 (0)	2 (6)	
**Complications overall (30-day Clavien-Dindo) n (%)**			0.37
1	–	1 (3)	
2	3 (12)	3 (9)	
3a	1 (4)		
3b	3 (12)		
4	–	1 (3)	
**Time from intervention to resection surgery (days)**			**<0.001**
median (min-max)	30 (1-63)	55 (11-258)	
**Cancer stage n (%)**			0.33
I	1 (4)	1 (3)	
II	17 (71)	17 (53)	
III	6 (25)	14 (44)	
**Adjuvant chemotherapy n (%)**			0.59
Yes	9 (38)	15 (47)	
**Local recurrence n (%)**			1
During follow-up	2 (8)	2 (6)	
**Distant metastasis n (%)**			0.49
During follow-up	3 (12)	7 (22)	
**Death n (%)**			0.08
During follow-up	16 (67)	14 (43)	
**Years of follow-up**			
Median (min-max)	18.5 (14-21)	14 (11-19)	**<0.001**

Bold values indicate statistical significance.

### 30-day complications

There were no differences in 30-day complications between the two groups, neither for complications related to the stent or stoma intervention nor for overall complications (**Table 1**). In the stent group, three patients had a slight abdominal pain one to two weeks after stent insertion. For one of them, the pathology report showed possible signs of microscopic perforation of the tumor, for the other two, there were no signs of perforation. None of these patients had any recurrence on follow-up. There were three stent failures where the patients continued to have colonic obstruction. One of the patients received a stent in the stent, which resolved the obstruction; the second patient was operated on with a diverting loop colostomy the day after. The third patient had a perforation in the ascending colon (the tumor was located in the sigmoid colon), and the patient was operated on with a subtotal colectomy with end ileostomy and a mucous colonic fistula. The total stent-related perforation rate was 1 in 24 (4.2%).

### Neoadjuvant and adjuvant treatment

All six rectal cancers in the stoma group received neoadjuvant treatment (RT or chemo/RT), none of the two rectal cancers in the stent group received neoadjuvant treatment. One patient in each group received adjuvant treatment. There was no recurrence among rectal cancers in the stoma group, and one recurrence in the stent group.

Among colon tumors, one patient in each group received neoadjuvant treatment. Among colon tumors stadium III, 79% (11/14) in the stoma and 75% (3/4) in the stent group received adjuvant treatment. There were 4 recurrences (29%) in the stoma, and 1 (25%) in the stent group. Among these, all but one in the stoma group had received adjuvant treatment.

Among colon tumors, stage II, 25% (3/12) in the stoma group and 29% (5/17) in the stent group received adjuvant treatment. No one in the stoma group and 4 out of 5 in the stent group had T4 tumors. There were 3 recurrences (25%) in the stoma and 1 (6%) in the stent group. None of the patients with recurrence in either group had received adjuvant treatment.

### Overall survival and recurrence-free survival

**Figure 3:**
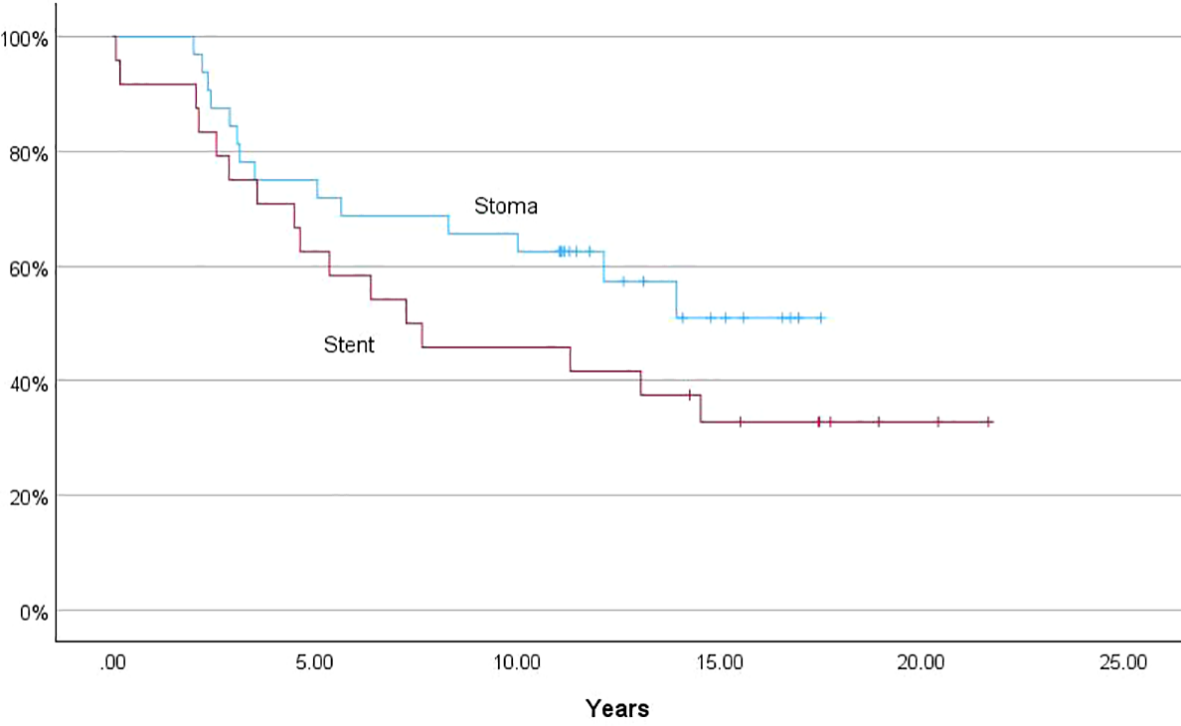
Kaplan-Meier curve, overall survival.

**Figure 4:**
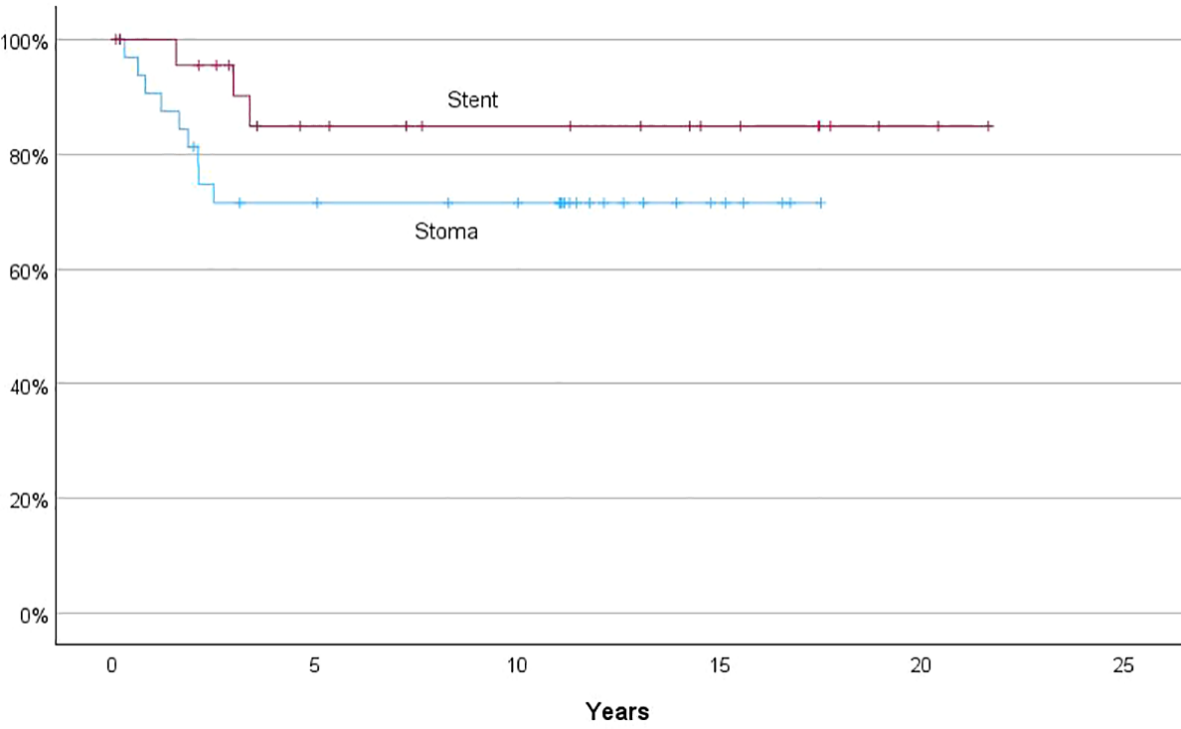
Kaplan-Meier curve, recurrence-free survival.

There was no significant difference in survival between patients in the stent group and the stoma group, neither regarding overall survival (**Figure 3**) nor recurrence-free survival (**Figure 4**).

### Cox regression analysis of survival

The proportional hazards assumption was tested with Shoenfeld residuals and was not violated. The hazard ratio for time to death (overall survival) for stent vs stoma was 0.99 (95% confidence interval [CI] 0.38-2.59) and 0.19 (CI 0.03-1.09) with adjustment for age, sex, calendar year, ASA-classification, and tumor stage. The corresponding unadjusted hazard ratio for time to recurrence or death (recurrence-free survival) was 1.64 (CI 0.80-3.38), and 0 0.42 (CI 0.12-1.57, **Table 2**.

**Table 2:** Hazard ratio (HR) with 95% confidence interval (CI) for overall survival and recurrence-free survival in patients treated with stent or deviating stoma.

	HR unadjusted (95% CI)	HR adjusted (95% CI)
Overall survival		
Stent (vs stoma)	1.64 (0.80-3.38)	0.99 (0.38-2.59)
Age (per year increase)	**1.06 (1.02-1.11)**	**1.06 (1.01-1.11)**
Sex (female vs male)	0.46 (0.21-1.05)	0.80 (0.33-1.90)
Calendar year (per year increase)	**0.87 (0.77-0.99)**	0.86 (0.71-1.02)
ASA-classification (ASA 3–4 vs ASA 1-2)	**2.24 (1.08-4.67)**	1.48 (0.66-3.54)
Tumor stage (vs stage I)		
II	2.37 (0.32-17.68)	2.44 (0.30-19.77)
III	1.86 (0.24-14.66)	2.76 (0.32-23.65)
Recurrence-free survival		
Stent (vs stoma)	0.42 (0.12-1.57)	0.19 (0.03-1.09)
Age (per year increase)	0.98 (0.94-1.03)	0.98 (0.92-1.03)
Sex (female vs male)	0.75 (0.23-2.50)	0.81 (0.24-2.82)
Calendar year (per year increase)	0.95 (0.79-1.14)	0.77 (0.58-1.02)
ASA-classification (ASA 3–4 vs ASA 1-2)	1.86 (0.59-5.85)	1.90 (0.52-6.99)
Tumor stage (vs stage I)*		
II	–	–
III	–	–

*Recurrence-free survival could not be estimated for tumor stage since there were no events in the stage I group.

Bold values indicate statistical significance.

## Discussion and conclusion

In this study, comparing stent insertion and stoma surgery for emergency decompression of malignant colonic obstruction as a bridge to elective surgery, we found similar overall survival as well as recurrence-free survival in both groups. The lack of difference between the groups may, however, be explained by a type II error. The Cox regression analysis indicated a continuous improvement in both overall survival and recurrence-free survival during the study period.

We specifically chose only to include patients who were operated on with a diverting stoma as a bridge to surgery in the surgical group. The reason for this was that there has been a trend during the last two decades, at least in Sweden, towards converting acute resections to elective ones, especially for left-sided tumors. During the study period, very few acute resections for such tumors were performed at our hospital (**Figure 2**). Furthermore, comparisons between different methods of decompressing malignant colonic obstruction have been poorly investigated, particularly with respect to long-term outcomes.

Even though acute resections have the advantage of treating the obstruction and the tumor right away, studies have shown that such an approach is associated with higher mortality and morbidity compared to elective surgery (4, 24–27). Ascanelli et al. reported higher mortality for patients operated on with acute resection compared to those operated on electively (28), and Jestin et al. showed that acute resections result in lower cancer-specific survival compared to those operated on electively (29).

However, comparing acute resection with stent as a bridge to surgery has diverging outcomes. In a meta-analysis from 2015 by Matsuda et al. based on 11 studies, of which two were RCTs, bridge to surgery achieved by stent insertion was similar to emergency tumor resection regarding overall survival, recurrence-free survival and recurrence (30). In 2016, Atukorale et al. reported a similar survival between patients treated with stent as a bridge to surgery and emergency tumor resection for malignant colonic obstruction in a systematic review of 49 articles (31). In 2017, a meta-analysis of 8 RCTs by Arezzo et al. on morbidity of colonic stenting versus emergency resection for malignant colonic obstruction reported a recurrence rate of 40% in the stent group compared to 27% in the emergency surgery group, based on four of the RCTs, this did not reach statistical significance (14). On the other hand, in a national registry study in the Netherlands of 1860 patients treated for malignant colorectal obstruction, Amelung et al. found a significantly lower mortality rate among patients treated with either decompressive colostomy or stent placement as a bridge to surgery than among those treated with emergency resection (32). The CReST trial, 2022, randomized 245 patients either to SEMS or acute resection with no differences in long time oncological outcome (33).

Rather few studies have compared long-term oncological results of either stent or stoma as a bridge to surgery. Axmarker reported in a nationwide registry-based study in Sweden during the period 2007-2009, where patients were followed until 2012, that long-term survival for either SEMS or stoma as a bridge to surgery was comparable (23). Amelung et al. found similar results in a retrospective cohort study following 88 patients during 3 and 5 years follow up (32).

In our study, the stent-related tumor perforation rate was 4.2% (one patient) and was not situated at the tumor site. This is a relatively low figure compared to other reports. Most stents (20 of 24 cases) were inserted by a single endoscopist, a colorectal surgeon with extensive experience. The knowledge of handling patients with colonic obstruction with risk of perforation may have contributed to refraining from placing stents in some patients with an increased risk of perforation.

In 2008, Van Hooft et al. had an early closure of their multi-center RCT (Stent-in 2 trial) due to a perforation rate of 13% in the stent group (34). This was one of the reasons why the use of stents as a bridge to surgery in curative patients has been questioned. In 2014, a long-term oncologic follow-up of the Stent-in 2 trial reported an insignificant benefit for emergency resection with better 4-year overall survival and 4-year disease-free survival (35). The perforation rate in the Van Hooft et al. study has been criticized, and in 2012, Zhang et al. showed a perforation rate of 1.2% in the stent group in a meta-analysis with 601 patients (36), and in 2014, van Halsema et al. had a perforation rate of 7.4% in their meta-analysis with 4086 patients (37). The reason for the difference in perforation rate is unknown, but the choice of stent type may be important and should be considered. In our study, Hanarostent was used in 23 of the 24 cases, while in the van Hooft trial, Wallflex stents were used exclusively. In a meta-analysis of 86 studies of a total of 4086 stent insertions, Wallflex stents had a perforation rate >10% compared to < 5% for Hanarostents (37).

Other complications than perforation related to stents are migration of the stent, failure of the stent to alleviate the obstruction, abdominal pain, and rectal bleeding. In this group, two patients had abdominal pain after stent insertion without having a perforation. Pain has been reported to be common but specific data on its prevalence is scarce (38). In this study, the technical success rate was 100% and the clinical success rate was 89%. In a large series by Meisner et al., a technical success rate of approximately 95% and a clinical success rate of approximately 91% were reported for stenting of acute malignant colonic obstruction (447 patients) (39), similar to our results.

There was a significant difference in the time from initial treatment to tumor resection between the two groups. This is partly explained by the fact that all six patients with rectal tumors in the stoma group received neoadjuvant treatment before surgery, which was not given to the two patients with rectal tumors in the stent group. All acute stoma operations but one were also done with open laparotomy, leading to a longer recovery and a reluctance to enter the abdomen too soon after the first operation.

The indication for adjuvant chemotherapy has slightly changed during the study period both in terms of indication and therapy given. There were no changes in indications for stage III tumors, but the indication to treat stage II tumors has, in some terms, been expanded over the study period. The lack of differences between the two groups in adjuvant treatment for stage II tumors may be explained by the higher proportion of Stadium II/T4 tumors in the stent group.

The present study has several limitations and some strengths. The fact that it is a single-center study results in a small sample size and potentially limits the external validity. The small sample size means that the results must be evaluated with great caution. The risk of type II error cannot be ruled out. On the other hand, studies with long-term oncological outcomes after SEMS and stoma as a bridge to surgery are few, and none, to our knowledge, has a follow-up time as long as in our study.

There is also a risk of selection bias when choosing between stent or stoma. To deal with this we conducted a separate survival analysis with only patients treated with stent insertion before 2011 and only patients treated with deviating stomas after 2011. This calendar cut-off was based on the treatment trends in Sweden previously mentioned.

A strength of this study is that the single-center design likely results in more standardized patient management than in a multicenter study. A limitation when comparing outcomes after stoma surgery and stenting for colon decompression is that stoma surgery can be performed safely by most colorectal surgeons. In contrast, colon stenting requires a skilled and dedicated endoscopist.

In conclusion, our data showed no difference in overall or recurrence-free survival between patients treated with stent or stoma as a bridge to surgery for malignant colonic obstruction, neither among all patients, in the analysis before and after 2011, nor in the propensity score matched groups. The improving survival over time seen in the Cox regression analysis is possibly due to better oncologic treatment. Although no conclusions can be drawn solely from these data and the risk of type II error cannot be ruled out, it is reasonable, based on the past and current literature, to believe that stents play a role in the treatment of colonic obstruction. Factors such as tumor characteristics and type of stent might be of importance for the successful use of stents and need further investigation, and an experienced endoscopist seems to be pivotal for patient outcome. We conclude that stent insertion appears to be a safe treatment strategy in selected patients at specialized centers.

## Data Availability

The original contributions presented in the study are included in the article/supplementary material. Further inquiries can be directed to the corresponding author.
